# An Adaptive Multi-Dimensional Vehicle Driving State Observer Based on Modified Sage–Husa UKF Algorithm

**DOI:** 10.3390/s20236889

**Published:** 2020-12-02

**Authors:** Zeyuan Luo, Zanhao Fu, Qiwei Xu

**Affiliations:** 1 Chongqing University-University of Cincinnati Joint Co-op Institute, Chongqing University, Chongqing 400030, China; luozy@mail.uc.edu (Z.L.); fuzo@mail.uc.edu (Z.F.); 2 State Key Laboratory of Power Transmission Equipment & System Security and New Technology, Chongqing University, Chongqing 400030, China

**Keywords:** vehicle driving state estimation, unscented Kalman filter, adaptive filter, Sage–Husa filter, vehicle system, robust estimation

## Abstract

An accurate vehicle driving state observer is a necessary condition for a safe automotive electronic control system. Vehicle driving state observer is challenged by unknown measurement noise and transient disturbances caused by complex working conditions and sensor failure. For the classical adaptive unscented Kalman filter (AUKF) algorithm, transient disturbances will cause the failure of state estimation and affect the subsequent process. This paper proposes an AUKF based on a modified Sage–Husa filter and divergence calculation technique for multi-dimensional vehicle driving state observation. Based on the seven-degrees-of-freedom vehicle model and the Dugoff tire model, the proposed algorithm corrects the measurement noise by using modified Sage–Husa maximum posteriori. To reduce the influence of transient disturbance on the subsequent process, covariance matrix is updated after divergence is detected. The effectiveness of the algorithm is tested on the double lane change and Sine Wave road conditions. The robustness of the algorithm is tested under severe transient disturbance. The results demonstrate that the modified Sage–Husa UKF algorithm can accurately detect transient disturbance and effectively reduce the resulted accumulated error. Compared to classical AUKF, our algorithm significantly improves the accuracy and robustness of vehicle driving state estimation. The research in this paper provides a reference for multi-dimensional data processing under changeable vehicle driving states.

## 1. Introduction

According to the released Global Status Report on Road Safety 2018 [1] by WHO, the annual road traffic deaths reached 1.35 million in 2016. An effective solution towards safe driving is a worldwide problem. The stability-control technique is one of the most important techniques in vehicle active security [2].

Since the 1990s, automotive electronics have developed rapidly. Many methods have been proposed to improve automotive safety, including anti-lock brake system (ABS) based on tire dynamic model [3], traction control system (TCS) based on slip ratio [4], electric stability program (ESP) based on measured rolling angle speed, acceleration, and yaw moment of a vehicle [5]. An accurate vehicle driving state is a precondition for these systems.

However, vehicle driving state cannot be obtained by vehicular sensors and requires additional sensors because of complex vehicle driving conditions, low accuracy, and high cost of vehicular sensors. Even if enough high-accuracy sensors are installed regardless of the cost, the changing accuracy and transient disturbance cannot be avoided in vehicle driving. Therefore, the accuracy and robustness of the vehicle driving state observer is a key problem in the study of the automotive electronic control system. It is also the prerequisite and essential condition of closed-loop control.

The observation of vehicle driving states refers to the process of using the data measured by sensors to estimate other parameters of the vehicle system. The estimated parameters are usually difficult or inaccurate to measure. Most studies in this field focus on obtaining more accurate estimation based on collecting multi-dimensional information by different information fusion technologies.

There are two kinds of commonly used observation methods. The first one is based on dynamics, including Kalman filter [6,7,8] and neural network [9,10]. As for the neural network, the internal mapping relation is closely related to training data. It is unrealistic to apply all working conditions training on neural networks. The second estimation method is based on kinematics [11]. Sensors are used to measure vehicle data and estimate target parameters by following kinematic relationships. This method requires sensors with high accuracy and the error will accumulate in the whole system, which is with low robustness.

When the target state is difficult to be directly detected by a sensor, there are two common options. Firstly, we can design an optimized sensing method and indirectly detect the state. Secondly, we can utilize some measurement-friendly states to estimate the target state. Automobile scientists keep working on using the second method to estimate vehicle driving states. Instead of hardware measurement, a proper and accurate estimation can not only save costs but also adaptive to different vehicles. Scientists are optimistic about the application of the Kalman filter because it is a process of estimation and correction and does not need to store or analyze raw observation data. The development of the Kalman filter and verification of real vehicles proves this idea.

In 1995, L.R.Ray et al. [12] developed an extended Kalman filter (EKF) to estimate non-linear vehicle driving state based on wheel angular velocity, longitudinal and lateral acceleration, and yaw rate. Although this method was not widely spread because of the immaturity, it promoted the usage of Kalman filter in vehicle driving state estimation.

Vehicle states change violently and non-linearly in normal running. Based on the unscented Kalman filter (UKF), Doumiati M et al. [13] designed a state estimator to solve problems when applying KF and EKF in nonlinear systems. They accurately estimated tire forces and sideslip angles. Their method proved the feasibility of using UKF soft sensing to replace previous high-cost measurements. However, they did not consider the driving states under realistic road conditions. Shengbo Eben Li et al. [14] uses linearly arrayed ultrasonic sensors to track moving objects around the vehicle, which focuses on the perception of the surrounding environment.

The application of GPS/IMU Fusion in vehicle state estimation improves the accuracy but relies on GPS signals and with high cost. When GPS signals face severe transient disturbances or loss, the filter will diverge [15], which is with low robustness.

Vincent Havyarimana et al. [16] proposed a hybrid approach combining square root unscented Kalman filter and particle filter to determine vehicle states. The approach considered noise as a limited gaussian kernel mixture and estimated states by sparse gaussian kernel model. It solves the problem of filter diverging when a transient disturbance or weak GPS signal occurs. However, it is still at a high cost due to the usage of the GPS/INS component.

Sage–Husa adaptive filtering is applied in the unscented transformation process. It can optimize the mean and the error covariance of the measurement and process noise. Sage–Husa adaptive filter is widely applied in target tracking. Based on SINS (Strapdown Inertial Navigation System), CNS (Celestial Navigation System), and GNSS (Global Navigation Satellite System), Shuqing Xu et al. [17] proposed a method to track the target in a multi-system and multi-model way. It focused on filter fusion and significantly improved tracking accuracy. To solve the undetermined measurement noise and system noise, a classical Sage–Husa adaptive filter was involved. Combining the traditional UKF and traditional Sage–Husa filter, Bin Huang et al. [18] proposed a method based on a four-degrees-of-freedom nonlinear vehicle dynamic model and a simplified Magic Formula tire model. Their method could effectively detect the change in vehicle state errors. To estimate process noise, Qingya Wu et al. [19] applied Sage–Husa sub-optimal maximum posteriori noise estimator to adaptive simplified spherical simplex unscented Kalman filter. Their approach was more efficient than AUKF and effectively improved computational accuracy. To further improve the Sage–Husa algorithm, Gang Li et al. [20] eliminated the estimation of process noise and the error correction term of measurement noise estimation. However, their algorithm was applied in an optimized system and did not consider transient disturbances.

In practical application, vehicle driving states are affected by many factors including working conditions and weather. The prior variables of the measurement noise are unknown. Although UKF and AUKF can track the changing state, the estimation results will lag true values because of the system’s internal unchangeable process noise [21]. When working conditions change drastically, the measurement information transmitted to the observer is inaccurate. The disturbance caused by the changing working condition will cause the failure of state estimation and affect the subsequent process. When prior noise statistics are unknown or inaccurate, the Sage–Husa estimator and Sage–Husa maximum posterior have shown their potentialities in estimating the measurement and process noise in various fields [22,23,24,25]. However, the Sage–Husa estimator and Sage–Husa maximum posterior will diverge after being disturbed.

Therefore, we propose an AUKF based on a modified Sage–Husa filter and divergence calculation technique for multi-dimensional vehicle driving state observation. The noise estimator is based on a modified Sage–Husa maximum posteriori estimation. Its purpose is to perform an online estimation on the mean and error covariance of measurement noise. Besides, we use the divergence calculation technique to modify the covariance matrix when a disturbance occurs. To evaluate the practical value of our method, we add a severe disturbance of 0.1 s in the robustness test. The result shows that the modified Sage–Husa UKF algorithm can effectively reduce the impact of disturbances.

The main contributions of our work are shown as follows:

1. We propose a modified Sage–Husa UKF algorithm. It effectively increases the filtering convergence speed and reduces the influence of transient disturbance on subsequent estimation.
2. We introduce divergence calculation to detect transient disturbances and solve the divergence problem of Sage–Husa maximum posteriori. Meanwhile, the introduction of divergence calculation technique reduces the amount of calculation.
3. We apply the modified Sage–Husa UKF algorithm on vehicle driving state estimation. The simulation result proves the robustness of our approach, which provides a reference for multi-dimensional data processing under changeable vehicle driving states.

As for the rest of this paper, Section 2 introduces the seven-degrees-of-freedom vehicle model and the Dugoff tire model. Section 3 introduces the proposed approach of vehicle driving state estimation based on the Modified Sage–Husa UKF algorithm. Section 4 introduces the simulation and robustness test results of our proposed approach. Section 5 concludes the paper.

## 2. Models

Our proposed method is based on the nonlinear seven-degrees-of-freedom dynamic vehicle model and the Dugoff tire model. The seven-degrees-of-freedom vehicle model contains all the variables we are concerned with. It was established to connect the measured variables from sensors with the obtained state variables. However, some new state variables were involved in the vehicle model, for example, the longitudinal and lateral tire forces. Since these variables could not be measured directly, the Dugoff tire model was involved to calculate and obtain these variables.

### 2.1. Seven-Degrees-of-Freedom Vehicle Model

**Figure 1:**
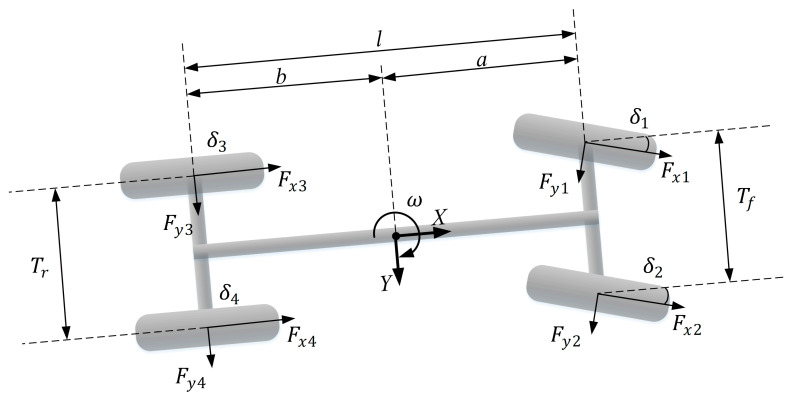
Schematic of seven-degrees-of-freedom vehicle model.

In this paper, we apply the estimation based on the seven-degrees-of-freedom vehicle model. The established seven-degrees-of-freedom dynamic vehicle model contains three longitudinal, lateral, and yaw degrees of freedom (DOFs), and four rotational DOFs of the wheels [26]. We can obtain longitudinal and lateral motion including speed and acceleration, yaw rate, side slip angle, and torque from this model. In Figure 1, the ISO coordinate system is set with the mass center of a vehicle as the origin, where the right part of the *x*-axis and the upper part of the *y*-axis is positive. The vehicle is longitudinal symmetry about the *x*-axis. The set of dynamic equations obtained from the model is shown as follows:

(1) $$\{\begin{array}{ll} \dot{u}= & a_{x}+v\omega \\ \dot{v}= & a_{y}+v\omega \\ \dot{\omega }= & \frac{\Gamma }{I_{z}}\\ \Gamma = & a(F_{x1}\sin\delta_{1}+F_{y1}\cos\delta_{1})-\frac{t_{f}}{2}(F_{x1}\cos\delta_{1}-F_{y1}\sin\delta_{1})+\\ & a\left(F_{x2}\sin\delta_{2}+F_{y2}\cos\delta_{2}\right)+\frac{t_{f}}{2}\left(F_{x2}\cos\delta_{2}-F_{y2}\sin\delta_{2}\right)-\\ & b\left(F_{x3}\sin\delta_{3}+F_{y3}\cos\delta_{3}\right)-\frac{t_{f}}{2}\left(F_{x3}\cos\delta_{3}-F_{y3}\sin\delta_{3}\right)-\\ & b\left(F_{x4}\sin\delta_{4}+F_{y4}\cos\delta_{4}\right)+\frac{t_{f}}{2}\left(F_{x4}\cos\delta_{4}-F_{y4}\sin\delta_{4}\right)+\\ \beta = & \frac{v}{u}\\ a_{x}= & \frac{1}{m}(F_{x1}\cos\delta_{1}-F_{y1}\sin\delta_{1}+F_{x2}\cos\delta_{2}-F_{y2}\sin\delta_{2}+\\ & F_{x3}\cos\delta_{3}-F_{y3}\sin\delta_{3}+F_{x4}\cos\delta_{4}-F_{y4}\sin\delta_{4}-F_{w})\\ a_{y}= & \frac{1}{m}(F_{x1}\sin\delta_{1}+F_{y1}\cos\delta_{1}+F_{x2}\sin\delta_{2}+F_{y2}\cos\delta_{2}+\\ & F_{x3}\sin\delta_{3}+F_{y3}\cos\delta_{3}+F_{x4}\sin\delta_{4}+F_{y4}\cos\delta_{4}) \end{array},$$

where m is the total mass of the vehicle; β is side slip angle; u is longitudinal speed; v is lateral speed; ω is yaw rate; $a_{x}$ is longitudinal acceleration; $a_{y}$ is lateral acceleration; Γ is torque around the *z*-axis; $I_{z}$ is car inertia around *z*-axis; δ is wheel steering angle; $F_{xi}$ is tire longitudinal force; $F_{yi}$ is tire lateral force; i=1, 2, 3, 4 represents the wheel at the front left, front right, rear left and rear right; $\delta_{i}$ represents the steering angle of the specific wheel responding to the wheel steer angle  δ; $F_{w}$ is wind resistance; a,b represent the distance from the mass center to the front axis and the back axis; $t_{f},t_{r}$ represent the distance of wheels at the front axis and back axis.

### 2.2. Dugoff Tire Model

**Figure 2:**
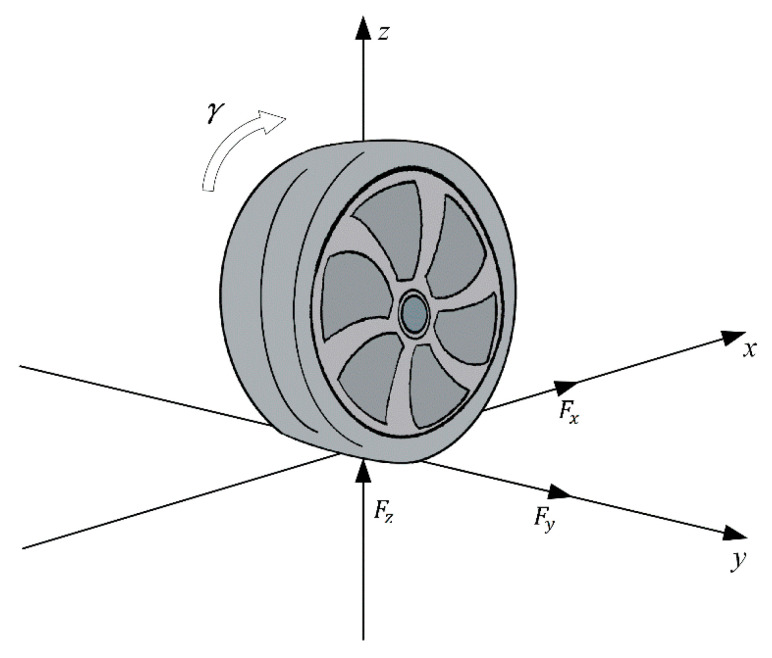
Schematic of Dugoff tire model.

Pacejka Magic Formula [27], Dugoff model [28], and Burckhardt model [29] is mostly used in these years to predict the behavior of tires. Comparing with the other two models, the Dugoff model is more suitable for UKF, and UKF can perfectly track the road adhesion coefficient. In this paper, we use the Dugoff tire model to simulate tire longitudinal ($F_{x}$) and lateral force ($F_{y}$). The schematic is shown in Figure 2. In this tire model, the tire longitudinal and lateral forces are closely related to parameters including longitudinal slip ratio λ, tire longitudinal ($C_{x}$) and lateral stiffness ($C_{y}$). 

The tire longitudinal and lateral force can be represented by Equations (2) and (3).

(2) $$F_{x}=C_{x}\frac{\lambda }{1-\lambda }f\left(L\right)$$

(3) $$F_{y}=C_{y}\frac{\tan\alpha }{1-\lambda }f\left(L\right),$$

where

(4) $$f\left(L\right)=\{\begin{array}{c} L\left(2-L\right),L<1\\ 1\;\;\;\;\;\;\;\;\;\;\;\;\;\;,L\geq 1 \end{array}$$

(5) $$L=\frac{\mu F_{z}}{\sqrt{C_{x}^{2}\lambda^{2}+C_{y}^{2}\tan^{2}\alpha \;}}\left(1-\lambda \right)\left(1-\varepsilon u\sqrt{C_{x}^{2}\lambda^{2}+C_{y}^{2}\tan^{2}\alpha }\right),$$

and μ is the current road adhesion coefficient; $F_{z}$ is the vertical load of each tire; L is the introduced boundary condition; ε is the speed influence factor. We obtain a road adhesion coefficient based on [30].

State variable x(t) is represented as $x\left(t\right)={\left[\begin{array}{ccc} v_{x} & v_{y} & \begin{array}{ccc} r & \Gamma & \begin{array}{cc} a_{x} & a_{y} \end{array} \end{array} \end{array}\right]}^{T}$. Observation variable y(t) is represented as $y\left(t\right)={\left[\begin{array}{ccc} a_{x} & a_{y} & r \end{array}\right]}^{T}$. Control input u(t)=[δ] is represented as. Based on the seven-degrees-of-freedom vehicle model and Dugoff tire model, state equation can be written as:

(6) $$X_{k+1}=f\left(X_{k},u_{k}\right)+\Lambda W_{k}={\left[\begin{array}{c} v_{x}\\ v_{y}\\ \begin{array}{c} r\\ 0\\ \begin{array}{c} 0\\ 0 \end{array} \end{array} \end{array}\right]}_{k-1}+{\left[\begin{array}{c} a_{x}+v_{y}r\\ a_{y}-v_{x}r\\ \begin{array}{c} \Gamma /I_{z}\\ 0\\ \begin{array}{c} 0\\ 0 \end{array} \end{array} \end{array}\right]}_{k-1}\cdot t+{\left[\begin{array}{c} 0\\ 0\\ \begin{array}{c} 0\\ \Gamma \\ \begin{array}{c} a_{x}\\ a_{y} \end{array} \end{array} \end{array}\right]}_{k}+\Lambda W_{k}.$$

System observation equation can be written as:

(7) $$Y_{k+1}=h\left(X_{k},u_{k}\right)+V_{k}=\left[\begin{array}{c} \begin{array}{ccc} 0 & 0 & \begin{array}{ccc} 0 & 0 & \begin{array}{cc} 1 & 0 \end{array} \end{array} \end{array}\\ \begin{array}{ccc} 0 & 0 & \begin{array}{ccc} 0 & 0 & \begin{array}{cc} 0 & 1 \end{array} \end{array} \end{array}\\ \begin{array}{ccc} 0 & 0 & \begin{array}{ccc} 1 & 0 & \begin{array}{cc} 0 & 0 \end{array} \end{array} \end{array} \end{array}\right]\cdot {\left[\begin{array}{c} v_{x}\\ v_{y}\\ \begin{array}{c} r\\ \Gamma \\ \begin{array}{c} a_{x}\\ a_{y} \end{array} \end{array} \end{array}\right]}_{k}+V_{k},$$

where k is the discrete time; Λ is the noise driving matrix; X(k) is the state at time k; Y(k)  is the observation matrix at time k; W(k)  is input white noise; and V(k) is observation noise.

## 3. Methods

### 3.1. Adaptive Unscented Kalman Filter (AUKF)

Compared to the unscented Kalman filter (UKF), AUKF is adaptive to process noise. UKF algorithm obtains the probability distribution of state variables by unscented transformation (UT). The nonlinear function is approximated based on probability density. The algorithm avoids the linearization of nonlinear systems [31].

The state and observation error equations can be represented as:

(8) $$x_{k}=f\left(x_{k-1}\right)+\Lambda W_{k}$$

(9) $$y_{k}=h\left(x_{k}\right)+V_{k},$$

where $x_{k}$ is the L-dimension state parameter of $t_{k}$ epoch; Λ is the noise driving matrix; $W_{k}$ is system noise matrix; $Q_{k},R_{k}$ is the covariance matrices of $W_{k},V_{k}$.

The description of AUKF is shown as follows [32,33]:

1. Sampling points construction. For L-dimension column-vector X, sampling points $\chi_{k-1}$ can be constructed by its estimation value ${\hat{\chi }}_{k-1}$ and variance $P_{k-1}$.
  (10) $$\{\begin{array}{ll} \chi_{i,k-1}={\hat{x}}_{k-1} & i=0\\ \chi_{i,k-1}={\hat{x}}_{k-1}+{\left[\sqrt{\left(L+\lambda \right)P_{k-1}}\right]}_{i} & i=1,\cdots ,L\\ \chi_{i,k-1}={\hat{x}}_{k-1}-{\left[\sqrt{\left(L+\lambda \right)P_{k-1}}\right]}_{i} & i=L+1,\cdots ,2L \end{array},$$
  where $\lambda =\alpha^{2}\left(L+\kappa \right)-L$, the selection of κ should ensure $\left(L+\kappa \right)P_{k-1}$ to be a positive semidefinite matrix, α affects the distribution of sampling points and should be within 0 to 1. ${\left[\sqrt{\left(L+\lambda \right)P_{k-1}}\right]}_{i}$ is the ith column of the matrix $\left[\sqrt{\left(L+\lambda \right)P_{k-1}}\right]$. In this paper, we assign α=0.01,κ=0.
2. Time update.
  (11) $$\chi_{i,k|k-1}=f\left(\chi_{i,k-1}\right),i=0,1,\cdots ,2L$$
  (12) $${\hat{x}}_{k|k-1}=\overset{2L}{\underset{i=0}{\sum }}\omega_{i}\chi_{i,k|k-1}$$
  (13) $$P_{k|k-1}=\sum_{i=0}^{2L}\omega_{i}{}^{c}\left(\chi_{i,k|k-1}-{\hat{x}}_{k|k-1}\right){\left(\chi_{i,k|k-1}-{\hat{x}}_{k|k-1}\right)}^{T},$$
  where $\omega_{i},\omega_{i}^{c}$ are the weights of sampling points. Equations (11)–(13) calculates the one-step prediction of Sigma points, the one-step prediction of system state variables, and the covariance matrix of system state variables. Another set of Sigma points needed to be constructed based on Equation (10).
3. Measurement update.
  (14) $${\hat{y}}_{i,k|k-1}=h\left(\chi_{i,k|k-1}\right)$$
  (15) $${\hat{y}}_{k}=\overset{2L}{\underset{i=0}{\sum }}\omega_{i}{\hat{y}}_{i,k|k-1}+{\hat{\sigma }}_{k-1}$$
  (16) $$P_{yy}=\overset{2L}{\underset{i=0}{\sum }}\left[\omega_{i}\left({\hat{y}}_{i,k|k-1}-{\hat{y}}_{k}\right){\left({\hat{y}}_{i,k|k-1}-{\hat{y}}_{k}\right)}^{T}\right]+R_{k}$$
  (17) $$P_{xy}=\overset{2L}{\underset{i=0}{\sum }}\left[\omega_{i}\left(\chi_{i,k|k-1}-{\hat{x}}_{k|k-1}\right){\left({\hat{y}}_{i,k|k-1}-{\hat{y}}_{k}\right)}^{T}\right]$$
  (18) $$K_{k+1}=P_{xy}P_{yy}^{-1}$$
  (19) $${\hat{x}}_{k}={\hat{x}}_{k|k-1}+K_{k}\left(y_{k}-{\hat{y}}_{k}\right)$$
  (20) $$P_{k}=P_{k|k-1}-K_{k}P_{yy}K_{k}^{T}.$$
  Equations (14)–(20) calculate the estimated observation variable, the mean of estimated observation variable, the covariance matrix of estimated observation variable, the covariance matrix of true observation variable, Kalman gain, state update, and covariance matrix update. $\sigma_{k}$ is the average value of noise $V_{k}$, and is usually assigned to be zero in AUKF.
4. Noise characteristics update.

The innovation is defined as:

(21) $$\varepsilon_{k}=y_{k}-{\hat{y}}_{k|k-1}.$$

The real-time estimated covariance matrix of the innovation sequence is calculated as:

(22) $$P_{\varepsilon \left(k\right)}=\frac{1}{M}\sum_{m=0}^{M-1}\left[\varepsilon \left(k-m\right)\varepsilon^{T}\left(k-m\right)\right]\;,$$

where $P_{\varepsilon \left(k\right)}$ is the covariance and M is the scale coefficient.

The update of the $R_{k}$ is

(23) $$R_{k}=P_{\varepsilon \left(k\right)}-\sum_{i=0}^{2L}\left[\omega_{i}\left(\chi_{i,k|k-1}-{\hat{y}}_{k|k-1}\right){\left(\chi_{i,k|k-1}-{\hat{y}}_{k|k-1}\right)}^{T}\right]\;.$$

This process estimates measurement noise and shows adaptiveness.

### 3.2. Divergence Calculation

Suppose there is a system described by the following state-space model

(24) $$X_{k+1}=\phi X_{k}+\Lambda W_{k}$$

(25) $$Y_{k}=HX_{k}+V_{k},$$

where k is the discrete time; ϕ is the state transition matrix; H is the observation matrix; Λ is the noise driving matrix; $X_{k}$ is the state at time k; $Y_{k}\;$ is the observation matrix at time k; $W_{k}\;$ is input white noise; $V_{k}$ is observation noise.

We assume that $W_{k}$ and $V_{k}$ are zero mean; the covariance matrices of $W_{k}$ and $V_{k}$ is $Q_{k}$ and $R_{k}$; $E\left[W_{k},V_{j}\right]=0$, where j represents random time.

Innovation is defined as

(26) $$\varepsilon_{k+1}=Y_{k+1}-{\hat{Y}}_{k+1|k}.$$

We have

(27) $${\hat{Y}}_{k+1}=H{\hat{X}}_{k+1|k}.$$

We can obtain Equation (28) if we substitute Equations (25) and (27) into Equation (26):

(28) $$\varepsilon_{k+1}=H\left(X_{k+1}-{\hat{X}}_{k+1|k}\right)+V_{k+1}.$$

The estimated variance of the measured value is

(29) $${\tilde{X}}_{k+1|k}=X_{k+1}-{\hat{X}}_{k+1|k}.$$

Then

(30) $$\varepsilon_{k+1}=H{\tilde{X}}_{k+1|k}+V_{k+1}.$$

The innovation covariance matrix can be expressed as

(31) $$Cov\left(\varepsilon_{k+1}\cdot \varepsilon_{k+1}{}^{T}\right)=HP_{k+1}H^{T}+R_{k+1}$$

where $P_{k+1}$ is the covariance matrix of the predicted error.

When the estimation converges, the innovation $\varepsilon_{k}$ is white noise that obeys the normal distribution. Hence, we can obtain Equation (32), where $\zeta_{xy}$ is the correlation coefficient, and the value is between zero and one. The closer $\zeta_{xy}$ approaches to zero, the weaker the correlation.

(32) $$\zeta_{xy}=\frac{Cov\left(X,Y\right)}{\sqrt{Var\left(X\right)}\cdot \sqrt{Var\left(Y\right)}}.$$

The correlation coefficient of innovation can be used to judge the divergence. The critical value for identifying the filter divergence in this paper is $\zeta_{0}=0.4$. If Equation (33) is satisfied, the filter diverges.

(33) $$\zeta_{0}\cdot \sqrt{Var\left(X\right)}\cdot \sqrt{Var\left(Y\right)}>Cov\left(X,Y\right).$$

Therefore, the discriminant formula can be obtained by substituting Equation (31) into Equation (33):

(34) $$\zeta_{0}\cdot Var\left(\varepsilon_{k+1}\right)<HP_{k+1}H^{T}+R_{k+1}.$$

Since $V_{k+1}$ and $H{\tilde{X}}_{k+1|k}$ are not related, then

(35) $$P\left\{\left|H{\tilde{X}}_{k+1|k}\right|\leq 2.5\left(HP_{k+1}H^{T}\right)\right\}=98.8\%$$

(36) $$P\left\{\left|V_{k+1}\right|\leq 2.5\left(R_{k+1}\right)\right\}=98.8\%.$$

When $\zeta_{0}=0.4$, there is at least a 97.61% probability for successful recognition, and the error rate is 0.04–2.39%.

### 3.3. Modified Sage–Husa Unscented Kalman Filter

Based on the traditional UKF, Sage–Husa algorithm, and divergence calculation technique, we propose a modified Sage–Husa UKF algorithm. The algorithm is designed to be more applicable in the field of vehicle driving state estimation than classical AUKF. The description of the proposed algorithm is shown as follows:

1. Sampling points construction and time update. These two stages are the same as the classical AUKF mentioned above.
2. Divergence calculation. Based on covariance matching, the filter is convergent if and only if
  (37) $$\zeta_{0}\cdot Var\left(\varepsilon_{k}\right)<HP_{k}H^{T}+R_{k},$$
  where $\zeta_{0}=0.4$ is the predetermined correlation coefficient; $R_{k}$ is the covariance matrix of $V_{k}$.
  If the filter diverges, we should correct the covariance matrix $P_{k|k-1}$ [34]:
  (38) $$P_{k|k-1}=\lambda_{k}\overset{2n}{\underset{i=0}{\sum }}\left[W_{i}^{c}\left(x_{k|k-1}^{i}-{\hat{x}}_{k|k-1}\right){\left(x_{k|k-1}^{i}-{\hat{x}}_{k|k-1}\right)}^{T}\right]+Q_{k-1}$$
  (39) $$\lambda_{k}=\{\begin{array}{ll} \lambda_{0} & \lambda_{0}>1\\ 1 & \lambda_{0}\leq 1 \end{array}$$
  (40) $$\lambda_{0}=\frac{tr\left[C_{0,k}-R_{k}\right]}{tr\left(\sum_{i=0}^{2L}\omega_{i}\left({\hat{y}}_{i,k|k-1}-{\hat{y}}_{k}\right){\left({\hat{y}}_{i,k|k-1}-{\hat{y}}_{k}\right)}^{T}\right)}$$
  (41) $$C_{0,k}=\{\begin{array}{ll} \varepsilon_{k}*\varepsilon_{k}^{T} & k=0\\ \frac{\rho C_{0,k}+\varepsilon_{k}*\varepsilon_{k}^{T}}{1+\rho } & k\geq 1 \end{array}$$
  where ρ is within zero to one and assigned to be 0.95. A large ρ will reduce the residual information effect before time k, and strengthen the effect of the current residual vector.
  If the filter is convergent, then
  (42) $$P_{k|k-1}=\overset{2L}{\underset{i=0}{\sum }}\left[\omega_{i}{}^{c}\left(\chi_{i,k|k-1}-{\hat{x}}_{k|k-1}\right){\left(\chi_{i,k|k-1}-{\hat{x}}_{k|k-1}\right)}^{T}\right]+Q_{k},$$
  which is the same as classical AUKF.
3. Measurement update is the same as classical AUKF.
4. Noise characteristics update [35].
  (43) $${\hat{R}}_{k}=\left(1-d_{k}\right){\hat{R}}_{k-1}+d_{k}\left(v_{k}v_{k}^{T}-H_{k}P_{k|k-1}H_{k}^{T}\right)$$
  (44) $${\hat{\sigma }}_{k}=\left(1-d_{k}\right){\hat{r}}_{k-1}+d_{k}\left[y_{k}-\overset{2n}{\underset{i=0}{\sum }}\left[\omega_{i}^{m}h\left(x_{i,k+1|k},u_{k}\right)\right]\right]$$
  (45) $$d_{k}=(1-b)(1-b_{k+1}),$$
  where $H_{k}$ is the transition matrix of the nonlinear equation measuring equation.$\;d_{k}\left({\hat{x}}_{k|k}-\overset{2L}{\underset{i=0}{\sum }}\omega_{i}{}^{m}f\left(x_{k|k-1}^{i}\right)\right)$ only has a small effect on the system and can be ignored.

## 4. Experiments and Analysis

The experiments are performed on CarSim and Simulink. We establish a simulation model based on the models and algorithms mentioned in Section 2 and Section 3. The Modified Sage–Husa UKF algorithm is tested on both double lane change and Sine Wave road conditions. We focus on five parameters (longitudinal speed, lateral speed, yaw rate, longitudinal acceleration, and lateral acceleration) because these parameters are sensitive to changes and more state variables can be further calculated based on these five parameters. We first present normal simulation tests to verify that the proposed approach can reach or even superior to the performance of classical AUKF. Since our approach mainly aims at the weak robustness and the slow adaptive speed of AUKF, we present a robustness test to compare the performances of two methods under severe disturbances.

### 4.1. Simulation Platform

CarSim released by MSC in 1996 is a vehicle dynamics simulation software. It can simulate the vehicle response to the road, air, and driver behavior in the real driving process. Carsim can simulate three to six times the speed of actual motion and allows users to manually set parameters. It can directly establish a Simulink model, which is friendly with MATLAB users. This paper verifies our proposed approach based on CarSim and Simulink co-simulation. Driving state data is provided by CarSim and the on-line estimation on the modified Sage–Husa UKF algorithm is performed on Simulink. 

We first set the road condition as double lane change in Carsim, with a maximum speed of 60 km/h. We then test the performance of the algorithm under Sine Wave road condition, with a maximum speed of 90 km/h. The parameters of the vehicle model are shown in Table 1.

We import the simulation data from CarSim to Simulink and co-simulate with the established Dugoff tire model and seven-degrees-of-freedom vehicle model.

### 4.2. Simulation Model

**Figure 3:**
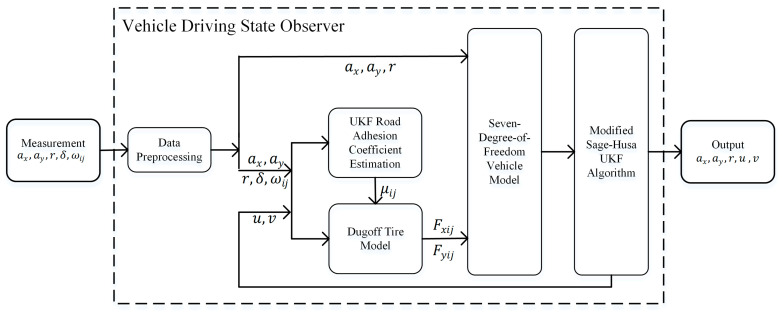
Schematic of the estimation process.

The established simulation model is shown in Figure 3. The output state variables of CarSim is time linear, including wheel steering angle δ, longitudinal acceleration $a_{x}$, lateral acceleration $a_{y}$, and yaw rate *r*. We can calculate the current tire longitudinal and lateral force $F_{xi}$ and $F_{yi}$ by inputting longitudinal and lateral acceleration, yaw rate, wheel steering angle, and estimated longitudinal and lateral speed into the Dugoff tire model. We can obtain current estimated longitudinal and lateral acceleration, yaw rate, and longitudinal and lateral speed by inputting $F_{xi}$, $F_{yi}$ and current measured accelerate and yaw rate into Vehicle Driving State Observer based on Modified Sage–Husa UKF algorithm. Longitudinal and lateral speed are regarded as output values and are involved with tire force calculation of the next moment. Here we suppose the acceleration only changes a little and the simulation result proves our hypothesis.

The main difference between the classical AUKF vehicle driving state estimation model and our model is the adaptive part. Instead of the classical AUKF algorithm, the proposed Vehicle Driving State Observer is based on the Modified Sage–Husa UKF algorithm.

**Figure 4:**
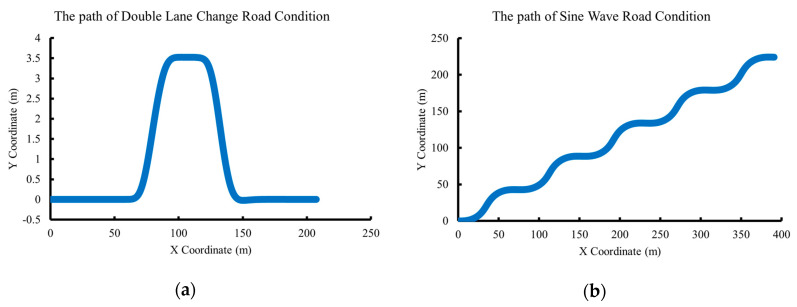
Schematic of testing road condition trajectory. (**a**) The trajectory of double lane change road condition. (**b**) The trajectory of Sine Wave road condition.

We compare the proposed Modified Sage–Husa UKF algorithm and classical AUKF based on double lane change and Sine Wave road conditions. The trajectories of the two road conditions are shown in Figure 4.

### 4.3. Simulation Results and Analysis

In the simulation, the initial measurement noise covariance matrix $R_{0}$ is

(46) $$R_{0}=\left[\begin{array}{ccc} 0.1 & & \\ & 0.1 & \\ & & 0.1 \end{array}\right]$$

The initial process noise covariance matrix $Q_{0}$ is

(47) $$Q_{0}=\left[\begin{array}{ccc} 0.1 & & \\ & 0.1 & \\ & & \begin{array}{ccc} 0.1 & & \\ & 0.1 & \\ & & \begin{array}{cc} 1 & \\ & 0.1 \end{array} \end{array} \end{array}\right]$$

The modified Sage–Husa UKF algorithm will adaptively iterate covariance matrix *R*. Hence the initial condition can be randomly selected within the proper range and the matrix *Q* must be positive definite. 

#### 4.3.1. Double Lane Change Road Condition

**Figure 5:**
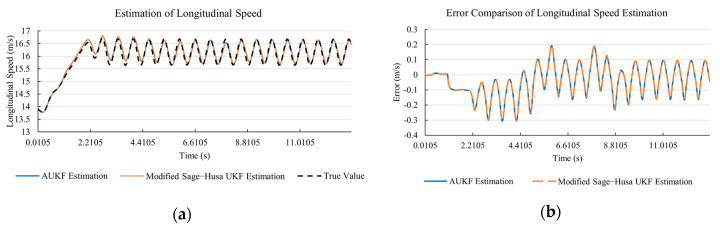
The simulation results of longitudinal speed under double lane change road condition. (**a**) The estimation comparison of longitudinal speed. (**b**) The error comparison of longitudinal speed estimation.

**Figure 6:**
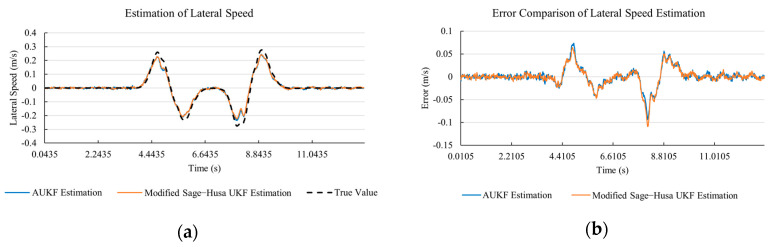
The simulation results of lateral speed under double lane change road condition. (**a**) The estimation comparison of lateral speed. (**b**) The error comparison of lateral speed estimation.

**Figure 7:**
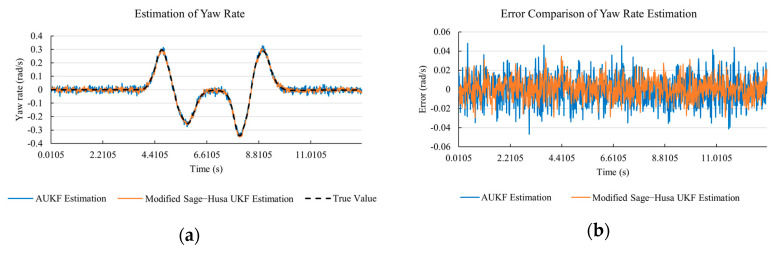
The simulation results of yaw rate under double lane change road condition. (**a**) The estimation comparison of yaw rate. (**b**) The error comparison of yaw rate estimation.

**Figure 8:**
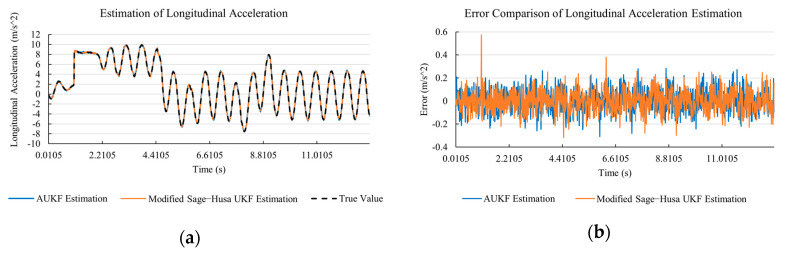
The simulation results of longitudinal acceleration under double lane change road condition. (**a**) The estimation comparison of longitudinal acceleration. (**b**) The error comparison of longitudinal acceleration estimation.

**Figure 9:**
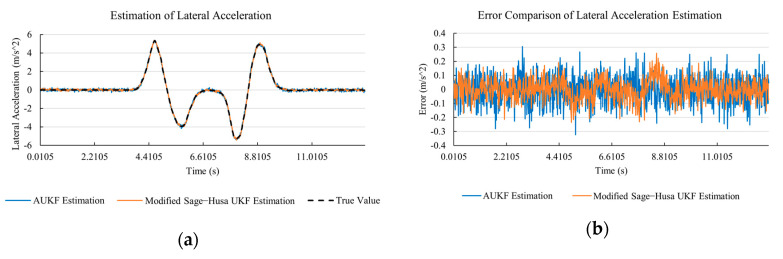
The simulation results of lateral acceleration under double lane change road condition. (**a**) The estimation comparison of lateral acceleration. (**b**) The error comparison of lateral acceleration estimation.

Figure 5a, Figure 6a, Figure 7a, Figure 8a, Figure 9a demonstrate the AUKF and modified Sage–Husa UKF algorithm estimation results. Figure 5b, Figure 6b, Figure 7b, Figure 8b, Figure 9b indicate the errors of two methods compared with true values. We apply RMSE (root mean square error) and MAE (mean absolute error) error evaluation and the results are shown in Table 2.

According to Table 2, the proposed Modified Sage–Husa UKF algorithm is better than AUKF in all five state variables and both two aspects. The RMSE values of longitudinal speed, lateral speed, yaw rate, longitudinal and lateral acceleration are improved by 0.71%, 2.4%, 30.7%, 4.9%, and 17.8% respectively compared to AUKF. The MAE values of these five state variables are improved by 0.68%, 2.4%, 30.0%, 3.4% and 19.7% respectively. 

#### 4.3.2. Sine Wave Road Condition

**Figure 10:**
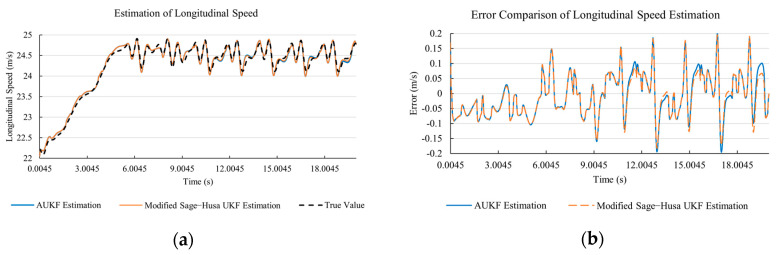
The simulation results of longitudinal speed under Sine Wave road condition. (**a**) The estimation comparison of longitudinal speed. (**b**) The error comparison of longitudinal speed estimation.

**Figure 11:**
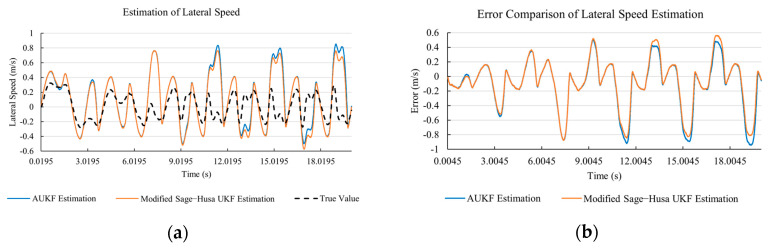
The simulation results of lateral speed under Sine Wave road condition. (**a**) The estimation comparison of lateral speed. (**b**) The error comparison of lateral speed estimation.

**Figure 12:**
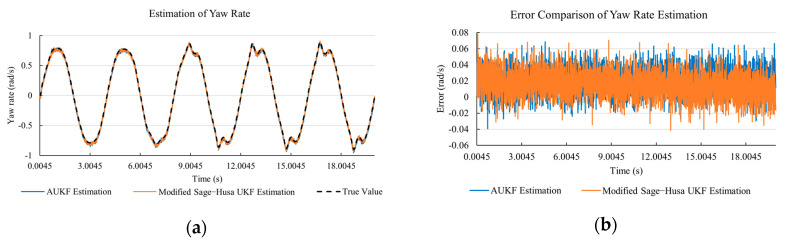
The simulation results of yaw rate under Sine Wave road condition. (**a**) The estimation comparison of yaw rate. (**b**) The error comparison of yaw rate estimation.

**Figure 13:**
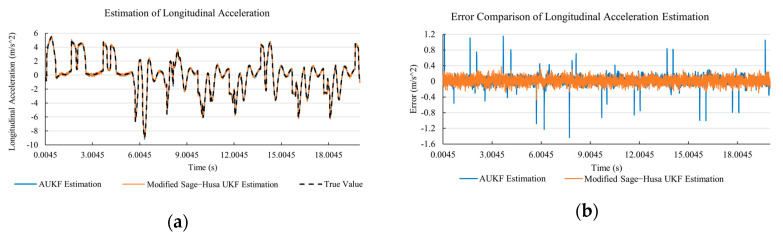
The simulation results of longitudinal acceleration under Sine Wave road condition. (**a**) The estimation comparison of longitudinal acceleration. (**b**) The error comparison of longitudinal acceleration estimation.

**Figure 14:**
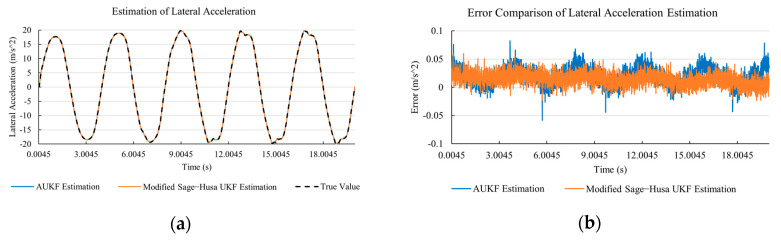
The simulation results of lateral acceleration under Sine Wave road condition. (**a**) The estimation comparison of lateral acceleration. (**b**) The error comparison of lateral acceleration estimation.

Figure 10a, Figure 11a, Figure 12a, Figure 13a, Figure 14a demonstrate the AUKF and modified Sage–Husa UKF algorithm estimation results. Figure 10b, Figure 11b, Figure 12b, Figure 13b, Figure 14b indicate the errors of two methods compared with true values. We apply RMSE (root mean square error) and MAE (mean absolute error) error evaluation and the results are shown in Table 3.

According to Table 3, the proposed Modified Sage–Husa UKF algorithm is better than AUKF in all five state variables and both two aspects. The RMSE values of longitudinal speed, lateral speed, yaw rate, longitudinal and lateral acceleration are improved by 1.19%, 0.63%, 1.21%, 53.45%, and 39.11% respectively compared to AUKF. The MAE values of these five state variables are improved by 1.97%, 2.55%, 2.38%, 40.06%, and 35.37% respectively.

#### 4.3.3. Discussion

Based on the results in Section 4.3.1 and Section 4.3.2, it can be seen that:

1. Our approach is with high accuracy and easy to converge. The MAE values near zero indicate small average deviations. Besides the involvement of the divergence calculation technique, we improve the filter as well. It should be noted that AUKF is so compact that even a little modification on the filter may result in lower accuracy. According to Table 2 and Table 3, in the normal road condition, the proposed Modified Sage–Husa UKF Algorithm can rival and even better than the performance of classical AUKF.
2. Our approach is adaptive to the changing system and remains excellent performance when the road condition changes. The initial conditions in Section 4.3.1 and Section 4.3.2 are the same. Since the initial matrices $R_{0}$ and $Q_{0}$ are set under double lane change road condition, AUKF performs undesirable in the Sine Wave road condition. Especially in Figure 13b and Figure 14b, the proposed approach demonstrates its superiority.
3. Our approach can update the covariance matrix. The introduction of the divergence calculation in our approach effectively improves the adaptivity and anti-interference ability. According to Figure 13b, the error curve of AUKF estimation is rough, which is with changeable extremum. This is caused by the comparatively more extreme road condition, which challenges the sensors and observers. AUKF performs undesirable because of its slow updating of the noise covariance matrix. The involvement of divergence calculation in the proposed method accelerates the process of convergence and performs desirable when the road condition changes.
4. It can be noticed that the estimation is undesirable in Figure 11a. This is because the Sine Wave road condition under 90 km/h is so extreme that most vehicle states reach their extreme values. Under this road condition, the direction of lateral speed changes with high frequency and small amplitude. The undesirable estimation of tire forces negatively affects the estimation of lateral speed. Nevertheless, the proposed approach tracks the changing trend and keeps convergent, which demonstrates its stability. Besides, it is comparatively unsafe to drive under 90 km/h in a real scenario. The test in Section 4.3.1 indicates that our approach can accurately estimate vehicle driving states under safe driving conditions.

### 4.4. Robustness Test

We simulate the change of measurement error or system error in the driving process. The purpose of this test is to simulate transient disturbances caused by an onboard processor failure in extreme conditions. 

#### 4.4.1. Double Lane Change Road Condition

We add a drastic disturbance with a length of 0.1 s at the 6.58th second. In this 0.1 s, longitudinal, lateral speed, yaw rate, longitudinal and lateral acceleration are set to be 15 m/s, 0.2 m/s, 0.2 rad/s, 15 m/s^2^, 15 m/s^2^ respectively when the real value is 16.67 m/s, 0.01 m/s, 0.01 rad/s, 0.21 m/s^2^, −0.12 m/s^2^ respectively. 

**Figure 15:**
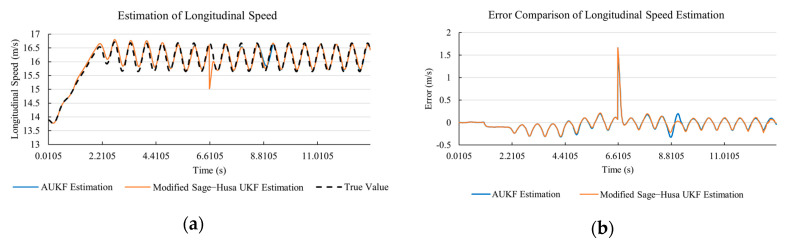
The simulation results of longitudinal speed in robustness test under double lane change road condition. (**a**) The estimation comparison of longitudinal speed in robustness test. (**b**) The error comparison of longitudinal speed estimation in robustness test.

**Figure 16:**
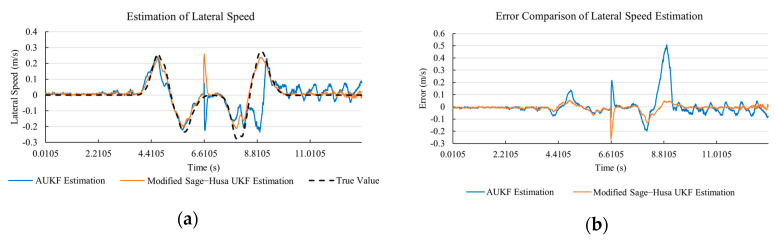
The simulation results of lateral speed in robustness test under double lane change road condition. (**a**) The estimation comparison of lateral speed in robustness test. (**b**) The error comparison of lateral speed estimation in robustness test.

**Figure 17:**
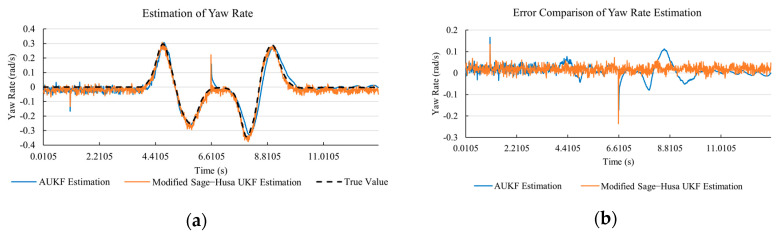
The simulation results of yaw rate in robustness test under double lane change road condition. (**a**) The estimation comparison of yaw rate in robustness test. (**b**) The error comparison of yaw rate estimation in robustness test.

**Figure 18:**
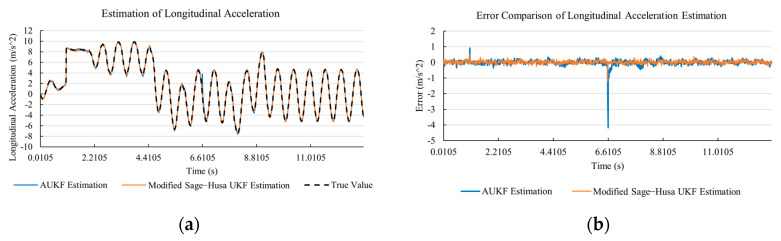
The simulation results of longitudinal acceleration in robustness test under double lane change road condition. (**a**) The estimation comparison of longitudinal acceleration in robustness test. (**b**) The error comparison of longitudinal acceleration estimation in robustness test.

**Figure 19:**
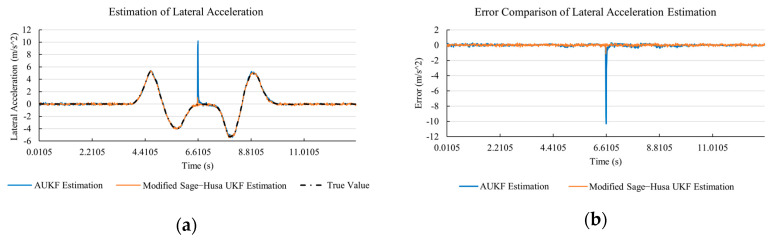
The simulation results of lateral acceleration in robustness test under double lane change road condition. (**a**) The estimation comparison of lateral acceleration in robustness test. (**b**) The error comparison of lateral acceleration estimation in robustness test.

Figure 15a, Figure 16a, Figure 17a, Figure 18a, Figure 19a demonstrate the AUKF and Modified Sage–Husa UKF algorithm estimation results. Figure 15b, Figure 16b, Figure 17b, Figure 18b, Figure 19b indicate the errors of the two methods compared to the true values. We apply RMSE and MAE error evaluation and the results are shown in Table 4.

The proposed Modified Sage–Husa UKF algorithm is better than AUKF in most state variables. The RMSE values of longitudinal speed, yaw rate, longitudinal and lateral acceleration are improved by 37.1%, 20.8%, 44.5%, and 76.1% respectively compared to AUKF. The MAE values of longitudinal speed, yaw rate, longitudinal and lateral acceleration are improved by 3.9%, 10.3%, 22.6%, and 19.4% respectively. As shown in Figure 15b, Figure 16b, Figure 17b, Figure 18b, Figure 19b, the Modified Sage–Husa UKF algorithm and AUKF both converge quickly to the true value after being disturbed. For the states shown in Figure 17a, Figure 18a, Figure 19a which exist measured values, the convergence speed of our approach is much faster than AUKF. In these three situations, our approach has improved convergence speed by 75%, 33%, and 67.2% respectively.

#### 4.4.2. Sine Wave Road Condition

We add a drastic disturbance with a length of 0.015 s at the 10th second. In this 0.015 s, longitudinal, lateral speed, yaw rate, longitudinal and lateral acceleration are set to be 22 m/s, 1 m/s, 0.2 rad/s, −17 m/s^2^, −8 m/s^2^ respectively when the real value is 24.58 m/s, 0.04 m/s, 0.02 rad/s, −4.03 m/s^2^, −0.27 m/s^2^ respectively. 

**Figure 20:**
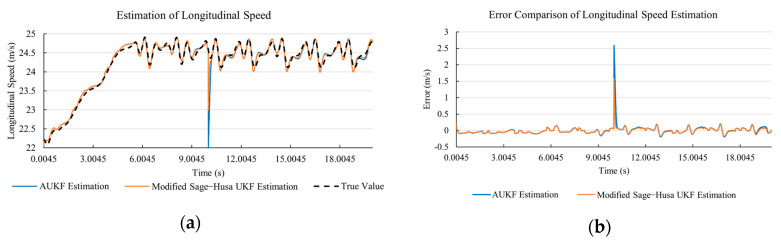
The simulation results of longitudinal speed in robustness test under sine wave road condition. (**a**) The estimation comparison of longitudinal speed in robustness test. (**b**) The error comparison of longitudinal speed estimation in robustness test.

**Figure 21:**
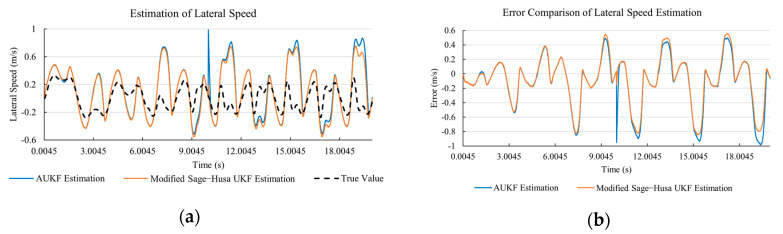
The simulation results of lateral speed in robustness test under sine wave road condition. (**a**) The estimation comparison of lateral speed in robustness test. (**b**) The error comparison of lateral speed estimation in robustness test.

**Figure 22:**
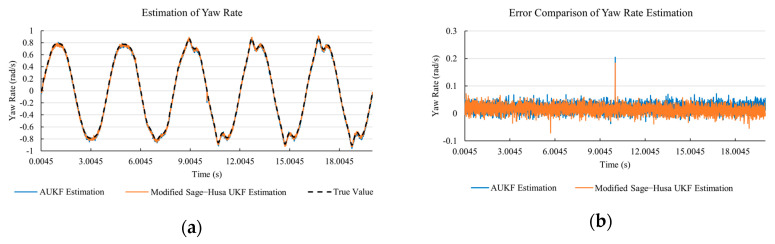
The simulation results of yaw rate in robustness test under sine wave road condition. (**a**) The estimation comparison of yaw rate in robustness test. (**b**) The error comparison of yaw rate estimation in robustness test.

**Figure 23:**
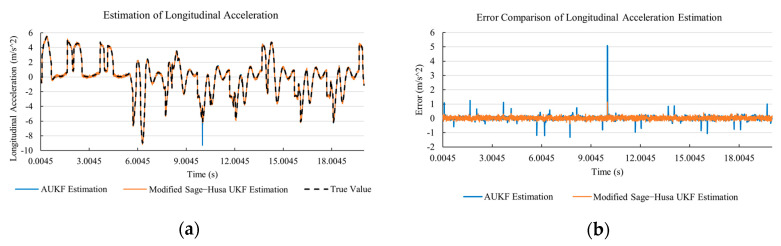
The simulation results of longitudinal acceleration in robustness test under sine wave road condition. (**a**) The estimation comparison of longitudinal acceleration in robustness test. (**b**) The error comparison of longitudinal acceleration estimation in robustness test.

**Figure 24:**
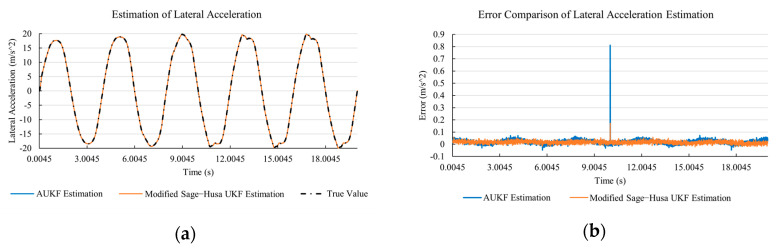
The simulation results of lateral acceleration in robustness test under sine wave road condition. (**a**) The estimation comparison of lateral acceleration in robustness test. (**b**) The error comparison of lateral acceleration estimation in robustness test.

Figure 20a, Figure 21a, Figure 22a, Figure 23a, Figure 24a demonstrate the AUKF and Modified Sage–Husa UKF algorithm estimation results. Figure 20b, Figure 21b, Figure 22b, Figure 23b, Figure 24b indicate the errors of the two methods compared to the true values. We apply RMSE and MAE error evaluation and the results are shown in Table 5.

According to Table 5, the proposed Modified Sage–Husa UKF algorithm is better than AUKF in all five state variables and both two aspects. The RMSE values of longitudinal speed, lateral speed, yaw rate, longitudinal and lateral acceleration are improved by 38.05%, 6.35%, 14.40%, 41.31%, and 35.90% respectively compared to AUKF. The MAE values of these five state variables are improved by 12.84%, 4.22%, 16.67%, 3.61%, and 21.56% respectively.

#### 4.4.3. Discussion

Based on the results in Section 4.4.1 and Section 4.4.2, it can be seen that:

1. The 57.27% MAE value improvement of lateral speed under double lane change road condition demonstrates that the transient disturbance impacts our approach less and does not affect the subsequent estimation.
2. Our approach has a better anti-interference ability than AUKF. According to Figure 15b, Figure 16b, Figure 17b, Figure 18b, Figure 19b, Figure 20b, Figure 21b, Figure 22b, Figure 23b, Figure 24b, the AUKF is sensitive to disturbances and takes a longer time at adapting.
3. As shown in Figure 9b, Figure 10b, Figure 11b, Figure 12b, Figure 13b, the accuracies of our approach are more stable when facing severe disturbances. This indicates that our approach can adapt to the most optimistic measurement noise covariance matrix.
4. As shown in the test under double lane change road condition, our approach can reflect the small state changes during the driving process, which is meaningful and important to the vehicle safety control system. This is because AUKF uses a windowing mask to eliminate the influence of state change on current estimation. The proportional coefficient *M* is set according to experiences. *M* cannot be determined or will be inaccurate if unknown changes occur.
5. According to Table 4, it can be noticed that in the lateral speed comparison, the RMSE value of the proposed approach is larger than that of the classical AUKF. There are two reasons. Firstly, the larger RMSE value here indicates that the proposed approach is more sensitive to the state change. It needs to be acknowledged that since we are performing on-line estimation when the disturbance is added, the system cannot distinguish whether the changing observation value is caused by a sudden change of vehicle state or just an exerted disturbance. The standard error is sensitive to the extremely large value or the extremely small value during the measurement. The proposed approach is sensitive to the state change, and the exerted disturbance is twenty times of the true value at that moment. Secondly, our approach is with a smaller MAE value under the circumstance of a larger RMSE value. This indicates our approach is with stronger robustness than classical AUKF. MAE value is the absolute value summation of the difference between the true value and the estimated value, which reflects the stability of the system.
6. According to Table 4 and Table 5, the proposed approach remains high accuracy and stability when the transient disturbance occurs, and it is with stronger robustness comparatively.

## 5. Conclusions

When the road condition changes frequently and drastically, traditional vehicle driving state estimation methods perform undesirable. The unknown measurement noise and transient disturbances will cause the failure of state estimation and affect the subsequent process. We propose an AUKF based on a modified Sage–Husa filter and divergence calculation technique to solve these problems. We mainly improve the classical AUKF algorithm in two aspects. First, we introduce the divergence calculation technique to detect transient disturbances and solve the divergence problem of Sage–Husa maximum posteriori. Second, we modify the covariance matrix when the transient disturbance occurs. 

Our approach can excellently face transient disturbances and has strong robustness through standard simulation tests and robustness tests under double lane change and Sine Wave road conditions. The proposed Modified Sage–Husa UKF algorithm fully satisfies the actual engineering precision requirements under simulation. The results of our approach rival the measurements obtained from high-cost and high-accuracy sensors. Besides, the simulation results show the feasibility of applying our approach to vehicle driving state estimation.

In our future work, we will first improve our algorithm, including the verification of more complicated working conditions and applications in a real car. Then we will add application scenarios for vehicle driving state estimation. The estimated data can combine with IoT (Internet of Things) and big data. For example, the vehicle driving states of a car getting out of control can share with surrounding cars. This will avoid secondary accidents. Data sharing could also share those repeated measuring state variables. It will save computational power and improve accuracy.

## Figures and Tables

**Table 1:** Parameters of the simulation vehicle model.

Parameter	Symbol	Value
Mass of the vehicle (kg)	m	1200
Height of mass center (m)	$h_{c}$	0.375
Distance from the front axis to the mass center (m)	a	1.455
Distance from the rear axis to the mass center (m)	b	1.195
Length of the front axis (m)	$T_{f}$	1.675
Length of the rear axis (m)	$T_{r}$	1.675
Inertia around the *z*-axis ($kg\cdot m^{2}$)	$I_{z}$	1.652

**Table 2:** Error evaluation under double lane change road condition.

State Variables	AUKF		Modified Sage–Husa UKF	
	RMSE	MAE	RMSE	MAE
Longitudinal speed	0.1124	0.0881	0.1116	0.0875
Lateral speed	0.0207	0.0124	0.0202	0.0121
Yaw rate	0.0140	0.0110	0.0097	0.0077
Longitudinal acceleration	0.0964	0.0755	0.0917	0.0729
Lateral acceleration	0.0926	0.0745	0.0761	0.0598

**Table 3:** Error evaluation under Sine Wave road condition.

State Variables	AUKF		Modified Sage–Husa UKF	
	RMSE	MAE	RMSE	MAE
Longitudinal speed	0.0671	0.0559	0.0663	0.0548
Lateral speed	0.3026	0.2161	0.3007	0.2106
Yaw rate	0.0248	0.0210	0.0245	0.0205
Longitudinal acceleration	0.2187	0.1333	0.1018	0.0799
Lateral acceleration	0.0381	0.0311	0.0232	0.0201

**Table 4:** Error evaluation in robustness test under double lane change road condition.

State Variables	AUKF		Modified Sage–Husa UKF	
	RMSE	MAE	RMSE	MAE
Longitudinal speed	0.1662	0.1030	0.1045	0.0990
Lateral speed	0.0863	0.0419	0.1018	0.0179
Yaw rate	0.0327	0.0233	0.0259	0.0209
Longitudinal acceleration	0.1883	0.1023	0.1045	0.0792
Lateral acceleration	0.4268	0.0953	0.1018	0.0768

**Table 5:** Error evaluation in robustness test under sine wave road condition.

State Variables	AUKF		Modified Sage–Husa UKF	
	RMSE	MAE	RMSE	MAE
Longitudinal speed	0.1490	0.0693	0.0923	0.0604
Lateral speed	0.3624	0.2561	0.3394	0.2453
Yaw rate	0.0257	0.0216	0.0220	0.0180
Longitudinal acceleration	0.1685	0.0803	0.0989	0.0774
Lateral acceleration	0.0312	0.0218	0.0200	0.0171
